# *fac*-Aceto­nitrile­tricarbon­yl(di­methyl­carbamodi­thio­ato-κ^2^ *S*,*S*′)rhenium(I): crystal structure and Hirshfeld surface analysis

**DOI:** 10.1107/S2056989017000755

**Published:** 2017-01-20

**Authors:** Sang Loon Tan, See Mun Lee, Peter J. Heard, Nathan R. Halcovitch, Edward R. T. Tiekink

**Affiliations:** a Research Centre for Crystalline Materials, School of Science and Technology, Sunway University, 47500 Bandar Sunway, Selangor Darul Ehsan, Malaysia; b Office of the Provost, Sunway University, 47500 Bandar Sunway, Selangor Darul Ehsan, Malaysia; c Department of Chemistry, Lancaster University, Lancaster LA1 4YB, UK

**Keywords:** crystal structure, rhenium, di­thio­carbamate, carbon­yl, Hirshfeld surface analysis

## Abstract

The title compound, [Re(C_3_H_6_NS_2_)(C_2_H_3_N)(CO)_3_], features an octa­hedrally coordinated Re^I^ atom within a C_3_NS_2_ donor set defined by three carbonyl ligands in a *facial* arrangement, an aceto­nitrile N atom and two S atoms derived from a symmetrically coordinating di­thio­carbamate ligand. In the crystal, di­thio­carbamate-methyl-H⋯O(carbon­yl) inter­actions lead to supra­molecular chains along [36-1]; both di­thio­carbamate S atoms participate in intra­molecular methyl-H⋯S inter­actions. Further but weaker aceto­nitrile-C—H⋯O(carbonyl) inter­actions assemble mol­ecules in the *ab* plane. The nature of the supra­molecular assembly was also probed by a Hirshfeld surface analysis. Despite their weak nature, the C—H⋯O contacts are predominant on the Hirshfeld surface and, indeed, on those of related [Re(CO)_3_(C_3_H_6_NS_2_)*L*] structures.

## Abstract 2

An octa­hedral *fac*-C_3_NS_2_ coordination geometry is found for the Re^I^ atom in the title compound; the di­thio­carbamate ligand forms symmetric Re—S bonds. In the crystal, supra­molecular layers are formed *via* weak C—H⋯O inter­actions.

## Chemical context   

The reaction between a secondary amine and carbon di­sulfide in the presence of an alkali metal hydroxide yields a class of ligands, the di­thio­carbamates, ^(−)^S_2_CN*R* *R*′. These ligands have long attracted the attention of coordination chemists owing to their high affinity for heavy-atom centres drawn from trans­ition metals, main group elements as well as lanthanides and actinides. The motivation for their study ranges across various disciplines and in the present time focuses upon their development as drugs (Hogarth, 2012 ▸; Bertrand & Casini, 2014 ▸), as chelating agents for the removal of toxic levels of metals in bio-remediation, *etc*. (Gallagher & Vo, 2015 ▸), as imaging/radio-pharmaceutical agents (Berry *et al.*, 2012 ▸) and as synthetic precursors for metal sulfide nanoparticles (Lewis *et al.*, 2015 ▸; Knapp & Carmalt, 2016 ▸). In terms of crystal engin­eering endeavours, di­thio­carbamates are not nearly as well studied as carboxyl­ates. This partly arises as a result of the greater chelating ability of di­thio­carbamate by virtue of the significant contribution of the ^(2−)^S_2_=CN^(+^ *^)^R* *R*′ canonical form to the electronic structure of the anion, *i.e*. with formal negative charges on each of the sulfur atoms. This has the consequence of reducing the Lewis acidity of the metal atom, often precluding additional donor atoms from entering the coord­ination sphere. Main group element di­thio­carbamate compounds are more likely to feature bridging ligands, often through secondary *M*⋯S inter­actions which may be mitigated by steric effects associated with the *R*,*R*′ groups or, in cases of organometallic derivatives, metal-bound substituents (Tiekink, 2006 ▸; Tiekink & Zukerman-Schpector, 2010 ▸). Another consequence of the tight chelating mode of the di­thio­carbamate ligands is the formation of aromatic *M*S_2_C chelate rings that can function as acceptors for C—H⋯ inter­actions, *i.e*. C—H⋯π(chelate) inter­actions (Tiekink & Zukerman-Schpector, 2011 ▸; Jotani *et al.*, 2016 ▸). As a result of the above, a very large number of crystal structure determin­ations have been reported in the literature, with the last systematic reviews published over a decade ago (Heard, 2005 ▸; Hogarth, 2005 ▸).

Reflecting the wealth of structural information on metal di­thio­carbamates, a search of the Cambridge Crystallographic Database (Groom *et al.*, 2016 ▸) for rhenium di­thio­carbamate structures reveals over 70 ‘hits’. One structure that attracted the attention of the authors was that of twofold symmetric, binuclear [(CO)_3_Re(S_2_CNEt_2_)]_2_, whereby each di­thio­carbamate ligand is μ_2_-tridentate, simultaneously chelating one Re^I^ atom while bridging a second (Flörke, 2014 ▸). The unusual feature of the structure is that the di­thio­carbamate ligands lie to one side of the mol­ecule and might be described as being *syn*. This arrangement is the same as that found in analogous, isoelectronic Pt^IV^ complexes (Heard *et al.*, 2000 ▸), but contradicts the observations seen in the overwhelming majority of the binary di­thio­carbamates of the zinc triad elements, a focus of present research, whereby binuclear mol­ecules with equal numbers of chelating and μ_2_-tridentate ligands lead to binuclear mol­ecules of the general formula, {*M*(S_2_CN*RR*’)_2_}_2_ (Cox & Tiekink, 2009 ▸; Tiekink, 2003 ▸; Tan *et al.*, 2016 ▸; Jotani *et al.*, 2016 ▸). This disparity lead to the attempted synthesis of the di­methyl­dithio­carbamate analogue of [(CO)_3_Re(S_2_CNEt_2_)]_2_, which when recrystallized from aceto­nitrile resulted in the isolation of mononuclear (CO)_3_Re(S_2_CNMe_2_)(N≡CMe), (I). Herein, the mol­ecular and crystal structures of (I) are described along with a detailed analysis of the self-assembly *via* a Hirshfeld surface analysis.
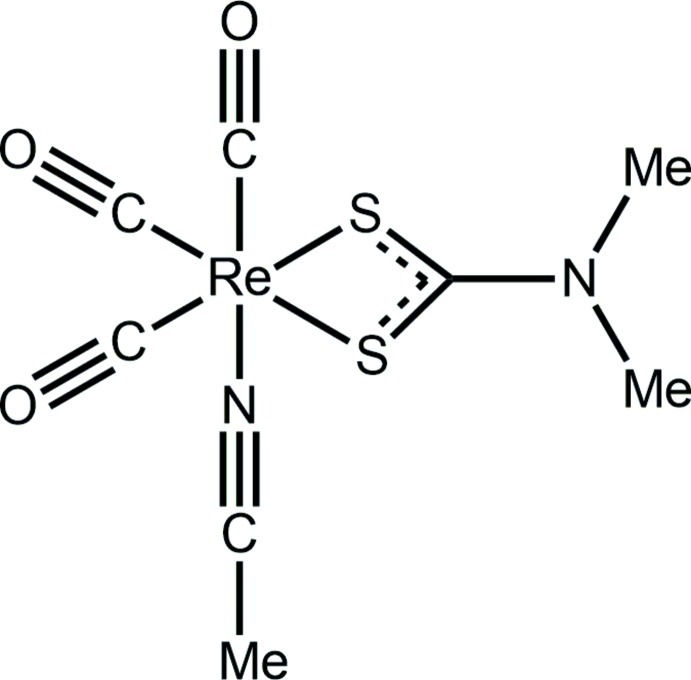


## Structural commentary   

**Figure 1:**
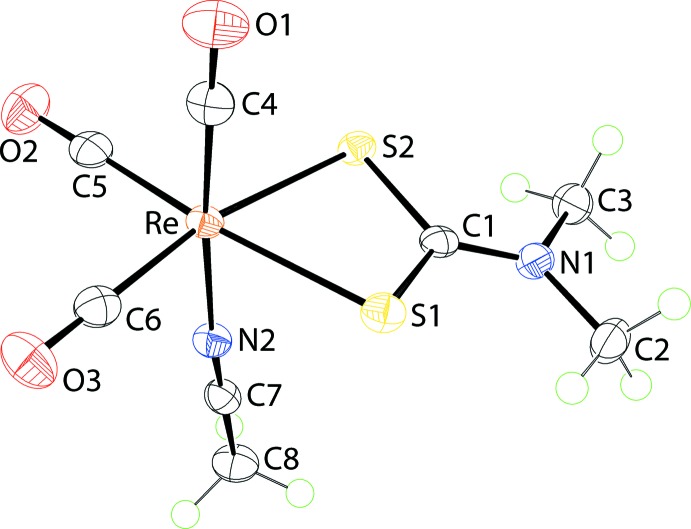
The mol­ecular structure of (I), showing the atom-labelling scheme and displacement ellipsoids at the 70% probability level.

The mol­ecular structure of (I) is shown in Fig. 1 ▸ and selected geometric parameters are collected in Table 1 ▸. The Re^I^ atom is coordinated by three *facially*-orientated carbonyl ligands, two di­thio­carbamate-S atoms and an aceto­nitrile-N atom. The di­thio­carbamate ligand is chelating in a symmetric mode with the difference between the long and short Re—S bond lengths being less than 0.01 Å. This mode of coordination is reflected in the equivalence of the associated C—S bond lengths and a relatively short C1—N1 bond length, Table 1 ▸, all pointing to a significant contribution of the ^(2−)^S_2_C=N^(+)^Me_2_ canonical form to the overall electronic structure of the di­thio­carbamate ligand. From the geometric data collected in Table 1 ▸, there is evidence that the shortest Re—CO bond length is formed by the carbonyl *trans* to the aceto­nitrile-N atom as opposed to those *trans* to the di­thio­carbamate-S atoms. However, the experimental errors do not allow definitive conclusions to be made. This point is discussed further in *Database survey* below.

## Supra­molecular features   

**Figure 2:**
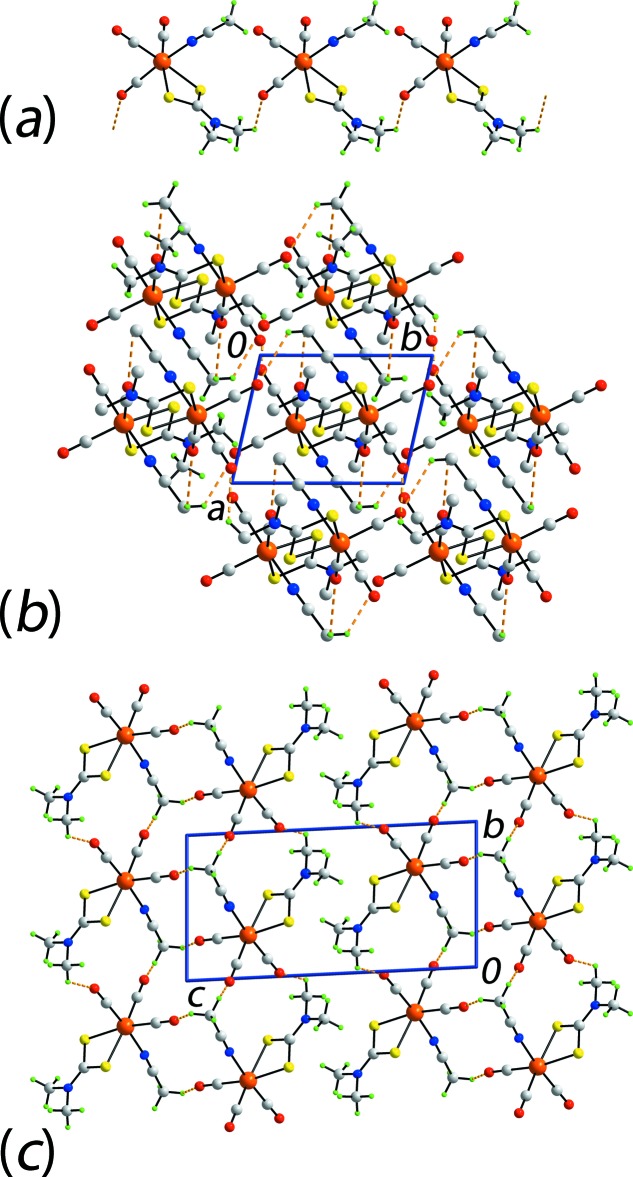
The mol­ecular packing in (I): (*a*) supra­molecular chain sustained by methyl-C—H⋯O(carbon­yl) inter­actions shown as orange dashed lines, (*b*) view of the supra­molecular layers in the *ab* plane with non-participating H atoms removed and (*c*) a view of the unit-cell contents in projection down the *a* axis.

Based on the standard criteria in *PLATON* (Spek, 2009 ▸), the most specific directional inter­action between mol­ecules in (I) is a di­thio­carbamate-methyl-H⋯O(carbon­yl) inter­action, Table 2 ▸. These lead to linear supra­molecular chains along [36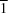], Fig. 2 ▸ *a*. Further searching for inter­molecular inter­actions reveals that the two remaining carbonyl-O atoms participate in weak C—H⋯O inter­actions just below the sum of the van der Waals radii, each with an aceto­nitrile-C—H atom, Table 2 ▸. The combination of these weak inter­actions leads to supra­molecular layers in the *ab* plane, Fig. 2 ▸ *b*. The two other potentially basic sites, namely the di­thio­carbamate-S atoms, form intra­molecular inter­actions with di­thio­carbamate-methyl-H atoms, Table 2 ▸. The layers stack along the *c* axis as shown in Fig. 2 ▸ *c*, *i.e*. without directional inter­actions between them.

## Hirshfeld surface analysis   

**Figure 3:**
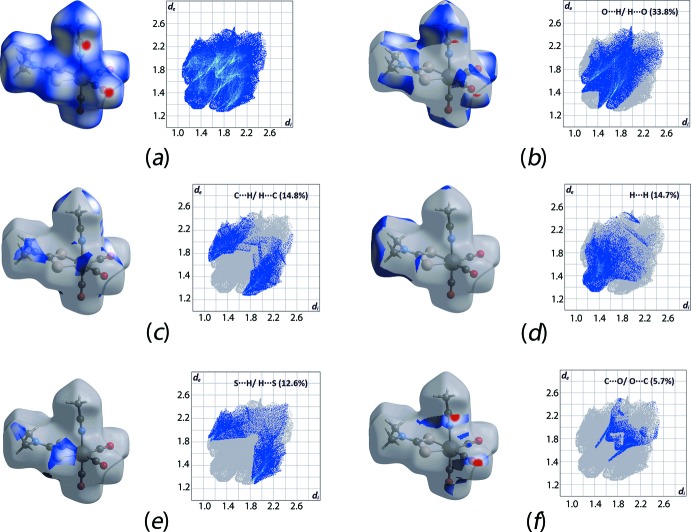
Hirshfeld *d*_norm_ surface and two-dimensional fingerprint plots for (I): (*a*) full plot, and those decomposed into (*b*) O⋯H/H⋯O, (*c*) C⋯H/H⋯C, (*d*) H⋯H, (*e*) S⋯H/H⋯S and (*f*) C⋯O/O⋯C contacts.

The protocols for the Hirshfeld surface analysis were as described recently (Yeo *et al.*, 2016 ▸). In general, the Hirshfeld surface of (I) features some close inter­action contacts as evidenced from the intense-red spots, Fig. 3 ▸ *a*, being indicative of *d*_norm_ contact distances shorter than the sum of van der Waals radii (McKinnon *et al.*, 2007 ▸). The combination of the *d*_i_ and *d*_e_, in inter­vals of 0.01 Å, resulted in the sparrow-like two-dimensional fingerprint plot. This has been decomposed into several close contacts as shown in Fig. 3 ▸ *b*–*f*. Specifically, the intense-red spots resulting from O⋯H/H⋯O as well as C⋯O/O⋯C contacts give bat- and scarab-shaped fingerprint profiles with corresponding *d*_e_ + *d*_i_ contact distances tipped at *ca* 2.5 and 3.0 Å, respectively; Fig. 3 ▸ *b* and *f*. These contact distances are approximately 0.25 Å shorter than the sum of the respective van der Waals radii (Batsanov, 2001 ▸) and constitute about 33.8 and 5.7%, respectively, of the overall Hirshfeld surface contacts for the mol­ecule. Other major contacts include C⋯H/H⋯C (14.8%), H⋯H (14.7%) and S⋯H/H⋯S (12.6%) which result in the pincer, bust sculpture and pincer forms of the respective decomposed fingerprint plots, despite the fact their contact distance are very close or equivalent to the sum of van der Waals radii with *d*_e_ + *d*_i_ values of 2.8, 2.4 and 2.9 Å, respectively; see Fig. 3 ▸ *c*–*e*.

## Database survey   

A series of eight closely related structural analogues with the formula [Re(CO)_3_(S_2_CNMe_2_)*L*], where *L* = ammonia (NH_3_) (1), pyridine (py) (2), imidazole (Im) (3), pyrazole (pz) (4), tri­phenyl­phosphine (PPh_3_) (5), 1,3,5-tri­aza-7-phosphaadamantane (PTA) (6), *t*-butyl isocyanide (tBuNC) (7) and cyclo­hexyl isocyanide (CyNC) (8) have been reported previously (Herrick *et al.*, 2009 ▸). The bond lengths about the Re^I^ atom in 1–8 and (I) are collated in Table 3 ▸; the numbering schemes correspond to that shown in Fig. 1 ▸. There are a few general observations that can be noted. Firstly, neither *d*(Re—S1) nor *d*(Re—S2) show major deviations in their respective bond lengths as evidenced from the mean difference of 0.005 Å for each. Despite the small differences, a trend is observed in that *d*(Re—S2) is generally longer than *d*(Re—S1). A consistent pattern is observed in the related *d*(Re—C5), *i.e. trans* to S1, and *d*(Re—C6), *i.e. trans* to S2, bond lengths for which the latter registers an average elongation of 0.005 Å. Secondly, the *d*(Re—*L*) bond lengths are found to consistently increase from C-donor ligands to N-donors, with a *ca* 0.10 Å or 5% increment, followed by P-donors with about a 0.26 Å or 12% increase, *cf*. the N-donor ligands. However, the observed trend deviates from expectation in that the *d*(*M*—*L*) bond length is anti­cipated to increase in the order N < C < P-donor type ligand by approximately 2.6 and 27.4%, respectively, based on their calculated covalent bond radii. Further, it is observed that *d*(Re—C4), *i.e*. with C4 *trans* to *L*, is marginally longer than *d*(Re—C5) and *d*(Re—C6) by *ca* 0.01–0.02 Å. Finally, *d*(C4≡O1) is generally shorter, by about 0.01 Å, *cf. d*(C5≡O2) and *d*(C6≡O3), *i.e*. with C5 and C6 *trans* to the S1 and S2 atoms, respectively. These observations show the presence of strong π-backbonding prevailing in the C-donor type ligands that result in shorter Re—*L* and longer Re—C4 bonds as well as shorter C4≡O1 bond lengths when compared to the other structural analogues. Further, these trends are clearly reflected in the blue shift of the νCO vibrational band for *L* = C-type donor ligands, with an average Δν = 180 cm^−1^, compared with those for N- and P-type donors (Herrick *et al.*, 2009 ▸). In the present study, ν(CO) for (I) was observed at 1883 cm^−1^.

**Figure 4:**
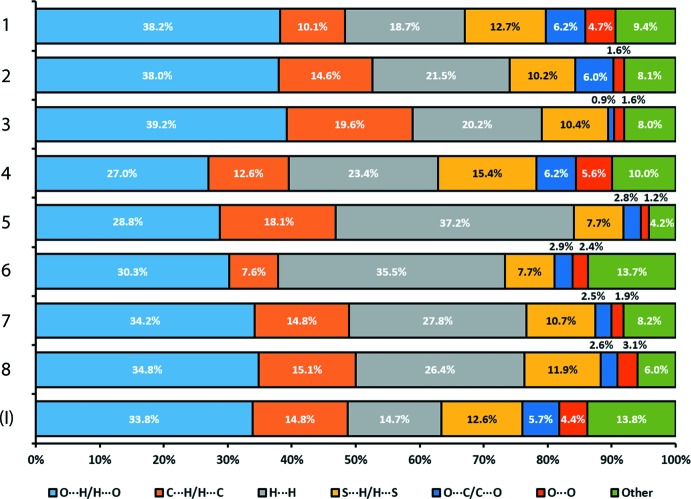
Percentage contributions of the different close contacts to the Hirshfeld surfaces of (I) and 1–8.

The mol­ecular packing in each of 1–8 was also studied through Hirshfeld surface analysis by calculating the relative composition of each inter­molecular close contact present in the structure using *Crystal Explorer* (Wolff *et al.*, 2012 ▸); Fig. 4 ▸. Generally, the inter­molecular close contacts are dominated by O⋯H/H⋯O, H⋯H, followed by either C⋯H/H⋯C or S⋯H/H⋯S contacts, with the exceptional cases being for 5 and 6, with hydrogen-rich P-donor ligands, for which the dominance is in the order H⋯H > O⋯H/H⋯O > C⋯H/H⋯C > S⋯H/S⋯H. These results highlight the relative importance of the C—H⋯O contacts in these structures despite their relatively weak nature.

## Synthesis and crystallization   

All chemicals and solvents were used as purchased without purification, and all reactions were carried out under ambient conditions. The melting point was determined using an Electrothermal digital melting point apparatus and was uncorrected. The IR spectra were obtained on a Perkin Elmer Spectrum 400 FT Mid-IR/Far-IR spectrophotometer from 4000 to 400 cm^−1^ (abbreviations: *vs*, very strong; *s*, strong). ^1^H NMR spectra were recorded at room temperature in DMSO-*d*_6_ solution on a Bruker AVANCE-400 MHz instrument.

Bromo­penta­carbonyl­rhenium(I) (0.25 mmol, 0.102 g) in acetone (10 ml) was added to sodium di­methyl­dithio­carbamate hydrate (0.25 mmol, 0.036 g) in acetone (10 ml). The resulting mixture was stirred and refluxed for 2 h. The filtrate was evaporated until a precipitate was obtained. The precipitate was recrystallized from its aceto­nitrile solution. Colourless blocks were obtained from the slow evaporation of the filtrate. Yield: 0.064 g, 60%; M.p. 478–479 K. IR (cm^−1^): 2009 (*s*), 1883 (*vs*). ^1^H NMR (in DMSO-*d*_6_): δ 3.21 (*s*, 6H, N–CH_3_), 2.07 (*s*, 3H, C–CH_3_).

## Refinement   

Crystal data, data collection and structure refinement details are summarized in Table 4 ▸. Carbon-bound H atoms were placed in calculated positions (C—H = 0.98 Å) and were included in the refinement in the riding-model approximation, with *U*_iso_(H) set to 1.2*U*_eq_(C). The maximum and minimum residual electron density peaks of 0.80 and 1.21 e Å^−3^ were located 0.87 and 0.91 Å, respectively, from the Re atom.

## Supplementary Material

Crystal structure: contains datablock(s) I, global. DOI: 10.1107/S2056989017000755/hb7651sup1.cif

Structure factors: contains datablock(s) I. DOI: 10.1107/S2056989017000755/hb7651Isup2.hkl

CCDC reference: 1527565

Additional supporting information:  crystallographic information; 3D view; checkCIF report

## Figures and Tables

**Table 1:** Selected geometric parameters (Å, °)

Re—S1	2.4956 (6)	Re—C5	1.924 (2)
Re—S2	2.5034 (6)	C1—S1	1.722 (2)
Re—N2	2.153 (2)	C1—S2	1.727 (2)
Re—C4	1.909 (3)	C1—N1	1.320 (3)
Re—C6	1.921 (3)		
			
S1—Re—C5	169.42 (7)	N2—Re—C4	175.53 (9)
S2—Re—C6	168.98 (7)		

**Table 2:** Hydrogen-bond geometry (Å, °)

*D*—H⋯*A*	*D*—H	H⋯*A*	*D*⋯*A*	*D*—H⋯*A*
C2—H2*B*⋯O1^i^	0.98	2.59	3.260 (3)	126
C8—H8*C*⋯O2^ii^	0.98	2.69	3.332 (3)	123
C8—H8*B*⋯O3^iii^	0.98	2.69	3.244 (3)	116
C2—H2*C*⋯S1	0.98	2.49	3.030 (2)	114
C3—H3*A*⋯S2	0.98	2.64	3.035 (2)	105

Symmetry codes: (i) 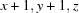; (ii) 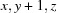; (iii) 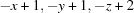.

**Table 3:** Selected bonding parameters (Å) for (I) and literature analogues [Re(CO)_3_(S_2_CNMe_2_)*L*]. *L* = ammonia (NH_3_) (1), pyridine (py) (2), imidazole (Im) (3), pyrazole (pz) (4), tri­phenyl­phosphine (PPh_3_) (5), 1,3,5-tri­aza-7-phosphaadamantane (PTA) (6), *t*-butyl isocyanide (tBuNC) (7) and cyclo­hexyl isocyanide (CyNC) (8) (Herrick *et al.*, 2009 ▸).

*L*	Re—S1	Re—S2	Re—C	C≡O	Re—C	C≡O	Re—C	C≡O	Re—*L*
			(*trans* to S1)		(*trans* to S2)		(*trans* to *L*)		
(1)	2.497 (2)	2.506 (2)	1.915 (7)	1.164 (8)	1.912 (6)	1.161 (7)	1.916 (7)	1.153 (9)	2.228 (5)
(2)	2.505 (2)	2.498 (1)	1.925 (6)	1.147 (7)	1.929 (5)	1.137 (7)	1.926 (5)	1.141 (7)	2.219 (4)
(3)	2.501 (2)	2.518 (3)	1.937 (7)	1.135 (8)	1.914 (7)	1.157 (9)	1.918 (7)	1.166 (8)	2.189 (6)
(4)	2.489 (4)	2.501 (4)	1.906 (14)	1.147 (17)	1.900 (14)	1.153 (17)	1.912 (13)	1.133 (16)	2.173 (10)
(5)	2.513 (3)	2.506 (3)	1.910 (10)	1.169 (13)	1.895 (10)	1.179 (12)	1.931 (10)	1.152 (12)	2.474 (3)
(6)	2.527 (5)	2.529 (4)	1.925 (15)	1.147 (19)	1.898 (16)	1.160 (20)	1.983 (18)	1.110 (20)	2.437 (5)
(7)	2.512 (3)	2.521 (2)	1.906 (7)	1.176 (9)	1.941 (8)	1.137 (9)	1.955 (8)	1.152 (9)	2.102 (7)
(8)	2.502 (2)	2.512 (2)	1.914 (9)	1.142 (12)	1.908 (10)	1.168 (12)	1.953 (9)	1.125 (11)	2.082 (9)
(I)	2.496 (1)	2.503 (1)	1.924 (2)	1.150 (3)	1.921 (3)	1.145 (3)	1.909 (3)	1.155 (3)	2.153 (2)

**Table 4:** Experimental details

Crystal data	
Chemical formula	[Re(C_3_H_6_NS_2_)(C_2_H_3_N)(CO)_3_]
*M*_r_	431.49
Crystal system, space group	Triclinic, *P* 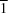
Temperature (K)	100
*a*, *b*, *c* (Å)	5.7442 (1), 7.5022 (1), 14.6644 (2)
α, β, γ (°)	91.496 (1), 95.517 (1), 102.371 (1)
*V* (Å^3^)	613.71 (2)
*Z*	2
Radiation type	Mo *K*α
μ (mm^−1^)	10.23
Crystal size (mm)	0.15 × 0.11 × 0.11

Data collection	
Diffractometer	Agilent SuperNova Dual Source diffractometer with an AtlasS2 detector
Absorption correction	Gaussian (*CrysAlis PRO*; Rigaku Oxford Diffraction, 2015 ▸)
*T*_min_, *T*_max_	0.371, 0.503
No. of measured, independent and observed [*I* > 2σ(*I*)] reflections	32146, 3244, 3153
*R*_int_	0.033
(sin θ/λ)_max_ (Å^−1^)	0.698

Refinement	
*R*[*F* ^2^ > 2σ(*F* ^2^)], *wR*(*F* ^2^), *S*	0.016, 0.035, 1.09
No. of reflections	3244
No. of parameters	148
H-atom treatment	H-atom parameters constrained
Δρ_max_, Δρ_min_ (e Å^−3^)	0.80, −1.21

Computer programs: *CrysAlis PRO* (Rigaku Oxford Diffraction, 2015 ▸), *SHELXS97* (Sheldrick, 2008 ▸), *SHELXL2014* (Sheldrick, 2015 ▸), *ORTEP-3 for Windows* (Farrugia, 2012 ▸), *DIAMOND* (Brandenburg, 2006 ▸) and *publCIF* (Westrip, 2010 ▸).

## supplementary crystallographic information

### Crystal data

**Table:**

[Re(C_3_H_6_NS_2_)(C_2_H_3_N)(CO)_3_]	*Z* = 2
*M**_r_* = 431.49	*F*(000) = 404
Triclinic, *P*1	*D*_x_ = 2.335 Mg m^−^^3^
*a* = 5.7442 (1) Å	Mo *K*α radiation, λ = 0.71073 Å
*b* = 7.5022 (1) Å	Cell parameters from 22533 reflections
*c* = 14.6644 (2) Å	θ = 3.0–29.4°
α = 91.496 (1)°	µ = 10.23 mm^−^^1^
β = 95.517 (1)°	*T* = 100 K
γ = 102.371 (1)°	Block, colourless
*V* = 613.71 (2) Å^3^	0.15 × 0.11 × 0.11 mm

### Data collection

**Table:**

Agilent SuperNova Dual Source diffractometer with an AtlasS2 detector	3244 independent reflections
Radiation source: micro-focus sealed X-ray tube, SuperNova (Mo) X-ray Source	3153 reflections with *I* > 2σ(*I*)
Mirror monochromator	*R*_int_ = 0.033
ω scans	θ_max_ = 29.7°, θ_min_ = 2.8°
Absorption correction: gaussian (CrysAlis PRO; Rigaku Oxford Diffraction, 2015)	*h* = −7→7
*T*_min_ = 0.371, *T*_max_ = 0.503	*k* = −10→10
32146 measured reflections	*l* = −20→20

### Refinement

**Table:**

Refinement on *F*^2^	0 restraints
Least-squares matrix: full	Hydrogen site location: inferred from neighbouring sites
*R*[*F*^2^ > 2σ(*F*^2^)] = 0.016	H-atom parameters constrained
*wR*(*F*^2^) = 0.035	*w* = 1/[σ^2^(*F*_o_^2^) + (0.0185*P*)^2^ + 0.3859*P*] where *P* = (*F*_o_^2^ + 2*F*_c_^2^)/3
*S* = 1.09	(Δ/σ)_max_ = 0.002
3244 reflections	Δρ_max_ = 0.80 e Å^−^^3^
148 parameters	Δρ_min_ = −1.21 e Å^−^^3^

### Special details

**Table:**

Geometry. All esds (except the esd in the dihedral angle between two l.s. planes) are estimated using the full covariance matrix. The cell esds are taken into account individually in the estimation of esds in distances, angles and torsion angles; correlations between esds in cell parameters are only used when they are defined by crystal symmetry. An approximate (isotropic) treatment of cell esds is used for estimating esds involving l.s. planes.

### Fractional atomic coordinates and isotropic or equivalent isotropic displacement parameters (Å^2^)

**Table:**

	*x*	*y*	*z*	*U*_iso_*/*U*_eq_	
Re	0.53372 (2)	0.29366 (2)	0.78801 (2)	0.01195 (4)	
S1	0.40857 (10)	0.56377 (8)	0.71937 (4)	0.01596 (12)	
S2	0.74400 (10)	0.37175 (8)	0.64780 (4)	0.01389 (11)	
O1	0.1152 (3)	0.0231 (3)	0.68068 (14)	0.0259 (4)	
O2	0.7673 (3)	−0.0198 (3)	0.85255 (14)	0.0258 (4)	
O3	0.2514 (3)	0.2703 (3)	0.95601 (13)	0.0267 (4)	
N1	0.6870 (4)	0.6973 (3)	0.58960 (14)	0.0156 (4)	
N2	0.8304 (4)	0.4965 (3)	0.85327 (14)	0.0151 (4)	
C1	0.6222 (4)	0.5632 (3)	0.64470 (16)	0.0132 (4)	
C2	0.5732 (5)	0.8540 (3)	0.58562 (18)	0.0203 (5)	
H2A	0.4691	0.8457	0.5280	0.031*	
H2B	0.6968	0.9672	0.5888	0.031*	
H2C	0.4773	0.8542	0.6374	0.031*	
C3	0.8671 (4)	0.6924 (3)	0.52607 (17)	0.0190 (5)	
H3A	0.9870	0.6289	0.5534	0.028*	
H3B	0.9456	0.8176	0.5137	0.028*	
H3C	0.7895	0.6277	0.4685	0.028*	
C4	0.2721 (4)	0.1247 (3)	0.72181 (17)	0.0174 (5)	
C5	0.6800 (4)	0.0972 (3)	0.82800 (17)	0.0169 (5)	
C6	0.3585 (4)	0.2794 (3)	0.89373 (17)	0.0176 (5)	
C7	0.9880 (4)	0.6097 (3)	0.88229 (16)	0.0155 (5)	
C8	1.1894 (5)	0.7539 (4)	0.91890 (18)	0.0211 (5)	
H8A	1.3395	0.7194	0.9075	0.032*	
H8B	1.1849	0.7720	0.9851	0.032*	
H8C	1.1795	0.8676	0.8888	0.032*	

### Atomic displacement parameters (Å^2^)

**Table:**

	*U*^11^	*U*^22^	*U*^33^	*U*^12^	*U*^13^	*U*^23^
Re	0.00998 (5)	0.01241 (5)	0.01239 (5)	0.00051 (4)	0.00076 (3)	−0.00140 (3)
S1	0.0137 (3)	0.0178 (3)	0.0177 (3)	0.0058 (2)	0.0031 (2)	−0.0009 (2)
S2	0.0137 (3)	0.0131 (3)	0.0157 (3)	0.0040 (2)	0.0036 (2)	−0.0003 (2)
O1	0.0174 (9)	0.0261 (10)	0.0292 (10)	−0.0028 (8)	−0.0038 (8)	−0.0079 (8)
O2	0.0241 (10)	0.0208 (9)	0.0329 (11)	0.0067 (8)	−0.0005 (8)	0.0043 (8)
O3	0.0248 (10)	0.0359 (11)	0.0200 (9)	0.0058 (9)	0.0078 (8)	−0.0001 (8)
N1	0.0167 (10)	0.0138 (9)	0.0162 (10)	0.0040 (8)	0.0001 (8)	−0.0002 (8)
N2	0.0146 (10)	0.0153 (10)	0.0152 (10)	0.0027 (8)	0.0023 (8)	−0.0009 (8)
C1	0.0119 (10)	0.0130 (10)	0.0133 (11)	0.0020 (9)	−0.0022 (8)	−0.0030 (8)
C2	0.0227 (12)	0.0163 (11)	0.0232 (13)	0.0073 (10)	0.0004 (10)	0.0038 (10)
C3	0.0194 (12)	0.0175 (12)	0.0190 (12)	0.0008 (10)	0.0037 (10)	0.0020 (10)
C4	0.0142 (11)	0.0192 (12)	0.0186 (12)	0.0025 (10)	0.0037 (9)	−0.0006 (10)
C5	0.0143 (11)	0.0154 (11)	0.0176 (12)	−0.0034 (9)	0.0010 (9)	−0.0027 (9)
C6	0.0164 (11)	0.0170 (11)	0.0189 (12)	0.0043 (10)	−0.0008 (9)	−0.0014 (9)
C7	0.0154 (11)	0.0179 (11)	0.0139 (11)	0.0050 (10)	0.0018 (9)	0.0002 (9)
C8	0.0171 (12)	0.0202 (12)	0.0228 (13)	−0.0012 (10)	−0.0004 (10)	−0.0049 (10)

### Geometric parameters (Å, º)

**Table:**

Re—S1	2.4956 (6)	N1—C3	1.463 (3)
Re—S2	2.5034 (6)	N2—C7	1.140 (3)
Re—N2	2.153 (2)	C2—H2A	0.9800
Re—C4	1.909 (3)	C2—H2B	0.9800
Re—C6	1.921 (3)	C2—H2C	0.9800
Re—C5	1.924 (2)	C3—H3A	0.9800
C1—S1	1.722 (2)	C3—H3B	0.9800
C1—S2	1.727 (2)	C3—H3C	0.9800
O1—C4	1.155 (3)	C7—C8	1.453 (3)
O2—C5	1.150 (3)	C8—H8A	0.9800
O3—C6	1.145 (3)	C8—H8B	0.9800
C1—N1	1.320 (3)	C8—H8C	0.9800
N1—C2	1.462 (3)		
			
S1—Re—C5	169.42 (7)	S1—C1—S2	114.23 (13)
S2—Re—C6	168.98 (7)	N1—C2—H2A	109.5
N2—Re—C4	175.53 (9)	N1—C2—H2B	109.5
C4—Re—C6	89.79 (10)	H2A—C2—H2B	109.5
C4—Re—C5	91.01 (10)	N1—C2—H2C	109.5
C6—Re—C5	91.30 (10)	H2A—C2—H2C	109.5
C6—Re—N2	93.43 (9)	H2B—C2—H2C	109.5
C5—Re—N2	92.03 (9)	N1—C3—H3A	109.5
C4—Re—S1	92.99 (8)	N1—C3—H3B	109.5
C6—Re—S1	98.50 (7)	H3A—C3—H3B	109.5
N2—Re—S1	83.47 (6)	N1—C3—H3C	109.5
C4—Re—S2	93.39 (7)	H3A—C3—H3C	109.5
C5—Re—S2	99.18 (7)	H3B—C3—H3C	109.5
N2—Re—S2	82.89 (5)	O1—C4—Re	179.1 (2)
S1—Re—S2	70.812 (19)	O2—C5—Re	179.5 (2)
C1—S1—Re	87.05 (8)	O3—C6—Re	179.1 (2)
C1—S2—Re	86.69 (8)	N2—C7—C8	179.7 (3)
C1—N1—C2	121.6 (2)	C7—C8—H8A	109.5
C1—N1—C3	121.8 (2)	C7—C8—H8B	109.5
C2—N1—C3	116.5 (2)	H8A—C8—H8B	109.5
C7—N2—Re	175.3 (2)	C7—C8—H8C	109.5
N1—C1—S1	122.93 (18)	H8A—C8—H8C	109.5
N1—C1—S2	122.84 (18)	H8B—C8—H8C	109.5
			
C2—N1—C1—S1	−2.5 (3)	Re—S1—C1—N1	−170.0 (2)
C3—N1—C1—S1	−179.31 (18)	Re—S1—C1—S2	10.24 (11)
C2—N1—C1—S2	177.28 (18)	Re—S2—C1—N1	170.0 (2)
C3—N1—C1—S2	0.5 (3)	Re—S2—C1—S1	−10.21 (11)

### Hydrogen-bond geometry (Å, º)

**Table:**

*D*—H···*A*	*D*—H	H···*A*	*D*···*A*	*D*—H···*A*
C2—H2*B*···O1^i^	0.98	2.59	3.260 (3)	126
C8—H8*C*···O2^ii^	0.98	2.69	3.332 (3)	123
C8—H8*B*···O3^iii^	0.98	2.69	3.244 (3)	116
C2—H2*C*···S1	0.98	2.49	3.030 (2)	114
C3—H3*A*···S2	0.98	2.64	3.035 (2)	105

Symmetry codes: (i) *x*+1, *y*+1, *z*; (ii) *x*, *y*+1, *z*; (iii) −*x*+1, −*y*+1, −*z*+2.
